# Comparison of quality control methods for automated diffusion tensor imaging analysis pipelines

**DOI:** 10.1371/journal.pone.0226715

**Published:** 2019-12-20

**Authors:** Seyyed M. H. Haddad, Christopher J. M. Scott, Miracle Ozzoude, Melissa F. Holmes, Stephen R. Arnott, Nuwan D. Nanayakkara, Joel Ramirez, Sandra E. Black, Dar Dowlatshahi, Stephen C. Strother, Richard H. Swartz, Sean Symons, Manuel Montero-Odasso, ONDRI Investigators, Robert Bartha

**Affiliations:** 1 Centre for Functional and Metabolic Mapping, Robarts Research Institute, University of Western Ontario, London, Ontario, Canada; 2 L.C. Campbell Cognitive Neurology Research Unit, Hurvitz Brain Sciences Research Program, Sunnybrook Research Institute, University of Toronto, Toronto, Ontario, Canada; 3 Rotman Research Institute, Baycrest Centre, Toronto, Ontario, Canada; 4 Department of Medicine, Division of Neurology, Sunnybrook Health Sciences Centre, and University of Toronto, Toronto, Ontario, Canada; 5 Ottawa Hospital Research Institute, Ottawa, Ontario, Canada; 6 Department of Medical Biophysics, University of Toronto, Toronto, Ontario, Canada; 7 Sunnybrook Health Sciences Centre, University of Toronto, Stroke Research Program, Toronto, Ontario, Canada; 8 Department of Medical Imaging, Sunnybrook Health Sciences Centre, Toronto, Ontario, Canada; 9 Department of Medicine, Division of Geriatric Medicine, Parkwood Hospital, University of Western Ontario, London, Ontario, Canada; 10 Department of Medical Biophysics, University of Western Ontario, London, Ontario, Canada; University of North Carolina at Chapel Hill, UNITED STATES

## Abstract

The processing of brain diffusion tensor imaging (DTI) data for large cohort studies requires fully automatic pipelines to perform quality control (QC) and artifact/outlier removal procedures on the raw DTI data prior to calculation of diffusion parameters. In this study, three automatic DTI processing pipelines, each complying with the general ENIGMA framework, were designed by uniquely combining multiple image processing software tools. Different QC procedures based on the RESTORE algorithm, the DTIPrep protocol, and a combination of both methods were compared using simulated ground truth and artifact containing DTI datasets modeling eddy current induced distortions, various levels of motion artifacts, and thermal noise. Variability was also examined in 20 DTI datasets acquired in subjects with vascular cognitive impairment (VCI) from the multi-site Ontario Neurodegenerative Disease Research Initiative (ONDRI). The mean fractional anisotropy (FA), mean diffusivity (MD), axial diffusivity (AD), and radial diffusivity (RD) were calculated in global brain grey matter (GM) and white matter (WM) regions. For the simulated DTI datasets, the measure used to evaluate the performance of the pipelines was the normalized difference between the mean DTI metrics measured in GM and WM regions and the corresponding ground truth DTI value. The performance of the proposed pipelines was very similar, particularly in FA measurements. However, the pipeline based on the RESTORE algorithm was the most accurate when analyzing the artifact containing DTI datasets. The pipeline that combined the DTIPrep protocol and the RESTORE algorithm produced the lowest standard deviation in FA measurements in normal appearing WM across subjects. We concluded that this pipeline was the most robust and is preferred for automated analysis of multisite brain DTI data.

## Introduction

Diffusion tensor imaging (DTI) is a well-established magnetic resonance imaging (MRI) technique, sensitive to the microstructural organization of cerebral tissue constituents [1,2]. This technique can be used to characterize healthy cerebral tissue microstructure as well as diverse subtle pathological and age-related alterations within the brain [1,3]. Diffusion tensor imaging can also be used to characterize primary water diffusion paths along axonal fibers to study structural connectivity and white matter (WM) integrity [4].

To effectively interpret brain DTI data a number of consecutive image processing steps must be performed including data conversions, artifact removal (such as eddy current (EC), motion, and gradient distortion corrections), and tensor fitting before diffusion metrics are calculated [5–11]. Recently, quality control (QC) procedures to remove outliers (e.g. distorted gradient volumes) from the analysis, modify voxel-wise diffusion tensor components, and harmonize data have also been identified as necessary steps in DTI analysis pipelines [5,8–10,12–16]. The latter step, is particularly relevant when analyzing large multisite and/or multiscanner DTI datasets [9,15–17], including those from the UK Biobank project [5,18–20] and the Ontario Neurodegenerative Disease Research Initiative (ONDRI) [21].

Implementing such complicated [11] processing requires automation to reduce unconscious user errors [22] and increase processing speed for large datasets [9,15–17]. Concerted efforts have been directed towards implementing fully automatic image processing pipelines for the analysis of brain DTI data [5–10,13]. For example, the Enhanced Neuroimaging Genetics though Meta-Analysis (ENIGMA) Consortium, a collaborative network of researchers working in the areas of neuroscience, genetics, and medicine, have analyzed neuroimaging data from thousands of subjects [9,23,24]. The ENIGMA DTI working group has created a well-known and widely accepted framework for region of interest (ROI) and tract-based analyses, and harmonization of DTI data from multiple sites [9,24]. In particular, the ENIGMA DTI working group focus has been on the analysis of high resolution multisite fractional anisotropy (FA) maps from healthy subjects to study the influence of genetic factors on the intersubject variabilities of WM microstructure [23,24]. Similarly, the Human Connectome Project (HCP), a consortium of institutions across the Unites States and Europe, aims to delineate brain connectivity and WM networks based on brain DTI analysis and tractography in a large numbers of healthy individuals [10,25].

Recently minimal preprocessing pipelines for automatic analysis of the HCP DTI data and other HCP MR imaging modalities were introduced [10]. In the HCP DTI preprocessing pipeline, essential image processing steps such as artifact (EC and gradient distortion) removal, surface generation, cross-modal linear registration, and transformation to a standard space were included [10]. This pipeline was designed to work with high quality DTI data produced by the HCP [10] and accordingly some QC procedures such as outlier detection and the removal of slice-wise and gradient-wise inconsistencies which are typically needed for conventional DTI data [13] were not included.

In the current study, we present, validate, and compare three fully automatic ENIGMA-based pipelines for processing brain DTI data by effectively connecting multiple image processing tools. We tested the performance of different automatic QC procedures as a supplementary image processing step added to the general ENIGMA recommended framework. For this purpose, two major types of QC procedures were considered. The first one is DTIPrep [13,26], which fully rejects diffusion weighted volumes along gradient directions significantly affected by gradient distortion artifacts [13]. The second one is Robust Estimation of Tensors by Outlier Rejection (RESTORE) [27]. RESTORE iteratively detects local (voxel-wise) outliers in the diffusion volumes and removes them from the final tensor estimation procedure [27]. Using these QC procedures, we devised three pipelines complying with the general ENIGMA framework. The first one used RESTORE as a QC procedure, the second one used the DTIPrep protocol, and the third one used a combination of both. The performance of the pipelines was validated and evaluated using “*ground truth*” and “*artifactual*” simulated diffusion weighted images (DWIs) generated based on the frameworks introduced in [28,29]. Pipelines were also applied to multi-site diffusion data from the ONDRI study to assess variance in normal tissue. We hypothesized that the third pipeline, which combined both QC procedures would produce optimal measurement precision by generating a smaller range of DTI parameter values in normal appearing WM.

## Materials and methods

### Simulated DWI data

POSSUM (Physics-Oriented Simulated Scanner for Understanding MRI) is an FSL toolbox that simulates MRI data by solving the Bloch and Maxwell equations. The toolbox produces realistic MR images and artifacts [28]. The simulated DWI data in this study were generated by an extension to POSSUM [30,31] called DW-POSSUM [28]. DW-POSSUM [29] effectively simulates DWI data and related artifacts such as eddy current induced artifacts. Here we adopted two major types of DWI datasets from [29,32]: a ground truth DWI dataset and four corresponding datasets with motion artefact, eddy current artefact, and thermal noise (Rician noise). Specifically, one dataset contained a large amount of motion at SNR = 20 (LM-20), a second dataset contained a large amount of motion at SNR = 40 (LM-40), a third dataset contained a small amount of motion at SNR = 20 (SM-20), and a fourth dataset contained a small amount of motion at SNR = 40 (LM-40). Motion artifacts were added to the simulated datasets by applying various degrees of rigid rotations to the geometric object (head), before the MR simulation began, in order to model instantaneous between-volume motions [28,29]. Detailed profiles of the head rotations used to generate motion artifacts in both the large and small motion scenarios were provide in reference [29]. POSSUM required two major inputs to simulate DWI datasets. First a realistic brain object was provided by a full-brain segmentation of high resolution T_1_- and T_2_-weighted images (matrix size: 260×311×260 and voxel size: 0.7 mm) of a single subject from the WU-Minn HCP dataset [25]. These images were segmented using the FSL Fast toolbox [33]. Second, a diffusion-weighted pulse sequence was simulated by a voxel-wise spherical harmonic fit (order n = 8) [34] to the b = 700 s/mm^2^ shell considering 32 gradient directions plus four b = 0 images [28,29]. The image acquisition was simulated using an echo-planar imaging (EPI) pulse sequence with TE = 109 ms and TR = 7500 ms and a matrix size of 72×68×55 (isotropic voxel size of 2.5 mm).

### ONDRI participants

We included 20 participants with VCI aged 60–85 years (40% female) available in the ONDRI database. ONDRI is a multi-site longitudinal research study of diverse neurodegenerative diseases including VCI [21,35]. ONDRI includes neuroimaging and genomics in addition to assessments of cognition as well as language, speech, gait, retinal imaging, and eye tracking [21,35]. The study was approved by the Ethics Review Boards at all participating institutions [21].

### Magnetic resonance imaging data acquisition

The acquisition of neuroimaging data for ONDRI participants has been previously described and was consistent with the Canadian Dementia Imaging Protocol (CDIP) [21]. Briefly, T_1_-weighted, T_2_-weighted, fluid-attenuated inversion recovery (FLAIR), T_2_*-weighted, positron density (PD)-weighted image, resting state functional MRI (fMRI), and diffusion MRI (dMRI) images were acquired from 11 different 3.0T scanners across Ontario. Scanner specific details are provided in Table 1.

**Table 1:** DTI data acquisition parameters for the different MRI scanners utilized in the current study.

Scan Type Parameter value	Parameter ranges used in evaluation of protocol compliance according to site and scanner type						
	HGH	SBH	TWH	RRI	SMH	TOH	TBR
Scanner Brand	GE	GE	GE	Siemens	Siemens	Siemens	Philips
Scanner Model	MR 750	MR 750	Signa HDxt	Prisma	Skyra	Tim Trio	Achieva
**DTI**							
TR	9000	9000	11700	9400	9400	9500	9400
TE	[81:90]	[80:95]	[105:110]	[62:66]	53	96	[94:98]
Flip	90	90	90	90	90	90	90
pixelBandwidth	3906.25	3906.25	3906.25	2055.0	[2055.0:2056.0]	1955.0	2056.0
Matrix Size	128x128	128x128	128x128	1152x1152	1152x1152	128x128	1152x1152
Voxel Size (in mm)	2x2x2	2x2x2	2x2x2	2x2x2	2x2x2	2x2x2	2x2x2
slice	2310	2310	2310	31	31	2170	31
**DTI_b0**								
TR	-	-	-	9400	9400	9500	9400
TE	-	-	-	[62:66]	39	96	[94:98]
Flip	-	-	-	90	90	90	90
Pixel Bandwidth	-	-	-	2055.0	[2055.0:2056.0]	1955.0	2056.0
Matrix Size	-	-	-	128x128	128x128	128x128	128x128
Voxel Size (in mm)	-	-	-	2x2x2	2x2x2	2x2x2	2x2x2
slice	-	-	-	70	70	70	70

HGH = Hamilton General Hospital, SBH = Sunnybrook Health Sciences Centre, TWH = Toronto Western Hospital, RRI = Robarts Research Institute, SMH = St. Michael’s Hospital, TOH = Ottawa Hospital, TBR = Thunder Bay Regional Hospital, TR = repetition time, TE = echo time.

For the current study, dMRI data were available and incorporated from seven different scanners/sites. The DTI processing pipelines utilized T_1_-weighted (voxel dimensions of 1.0×1.0×1.0 mm^3^), T_2_-weighted images (0.9375×0.9375×3.0 mm^3^), and dMRI data. The details of the acquisition parameters and pulse sequences for the DTI data across the seven scanners used for analysis are provided in Table 1. All the ONDRI DTI data used in this study were produced with 30 different gradient directions (with b = 1000 s/mm) with voxel dimensions of 2.0×2.0×2.0 mm^3^. The DTI data produced on Siemens and Philips scanners were acquired with one b_0_ volume, while in the case of GE scanners, three b_0_ volumes were acquired. On the Siemens and Philips scanners, two additional b_0_ images were acquired with phase encoding reversed. These were utilized for EPI distortion corrections in the processing pipelines. The simulated DWI data used in this study were comparable to the ONDRI data in terms of the number of the gradient directions as well as b-value.

### Segmentation of cerebral tissues and vascular lesions

The procedures used to segment regions of interest have been detailed previously (Semi-Automatic Brain Region Extraction (SABRE) [36], Lesion Explorer [37,38], and Fuzzy Lesion EXtractor (FLEX) [39]), including a scan-rescan reliability analysis [40]. Briefly, interleaved proton density (PD), T_2_-weighted images, and FLAIR images were co-registered to the T_1_-weighted image, and a PD-T_2_ based mask was automatically generated and manually edited. Using this mask, the T_1_-weighted image was segmented using a multi-feature histogram method [41] to generate GM, WM, and cerebrospinal fluid (CSF) regions, and ventricular CSF (vCSF) was manually relabeled. WM hyperintensities (WMHs) and lacunae were also automatically identified using FLEX (FLAIR-based) and Lesion Explorer along with manual edits. Each lesion type was further subdivided into a region-based class (periventricular or deep) by an automated algorithm [37–39]. Lesion Explorer was also used to capture enlarged perivascular spaces (PVS) [38,42], and cortical strokes were manually traced. A combination of these tissue segmentation methods produced ten different tissue classes including normal appearing WM (NAWM), which was used in the current study to evaluate DTI processing pipeline performance.

### Ground truth DTI metrics

The ground truth DTI metrics were calculated by rigidly registering the (artifact-free) simulated DTI data to the high resolution T1-weighted image and then tensor fitting to calculate DTI values at each voxel. Registering the DTI data to the T1 space is part of each pipeline as recommended by ENIGMA framework.

### Automated DTI processing pipelines

Three different fully automated pipelines were designed for processing and analysis of the brain DWIs. All the pipelines complied with the general ENIGMA DTI framework, a well-established methodology for processing diffusion data [9,24]. In the proposed pipelines we effectively used and optimized seven different image processing software packages including 3D Slicer [43], FSL [44], Ants [45], Camino [46], DTIPrep [13], MRIcron [47], and DTI-TK [48]. In all pipelines, automated QC procedures were added to the ENIGMA DTI framework, allowing detection of outlier data for removal prior to calculation of the diffusion tensor. These QC procedures are essential in multisite studies, where data is acquired on typical scanners available in healthcare centers [21]. All the proposed pipelines were automated using Bash scripts that are executable on Linux-based operating systems. All analyses were performed on a commodity computer work station with an Intel Core i7-6800k @3.4 GHz × 12 CPU, 16 GB RAM, GeForce GTX 1050 Graphics card, and a Linux Ubuntu 64-bit operating system.

**Fig 1:**
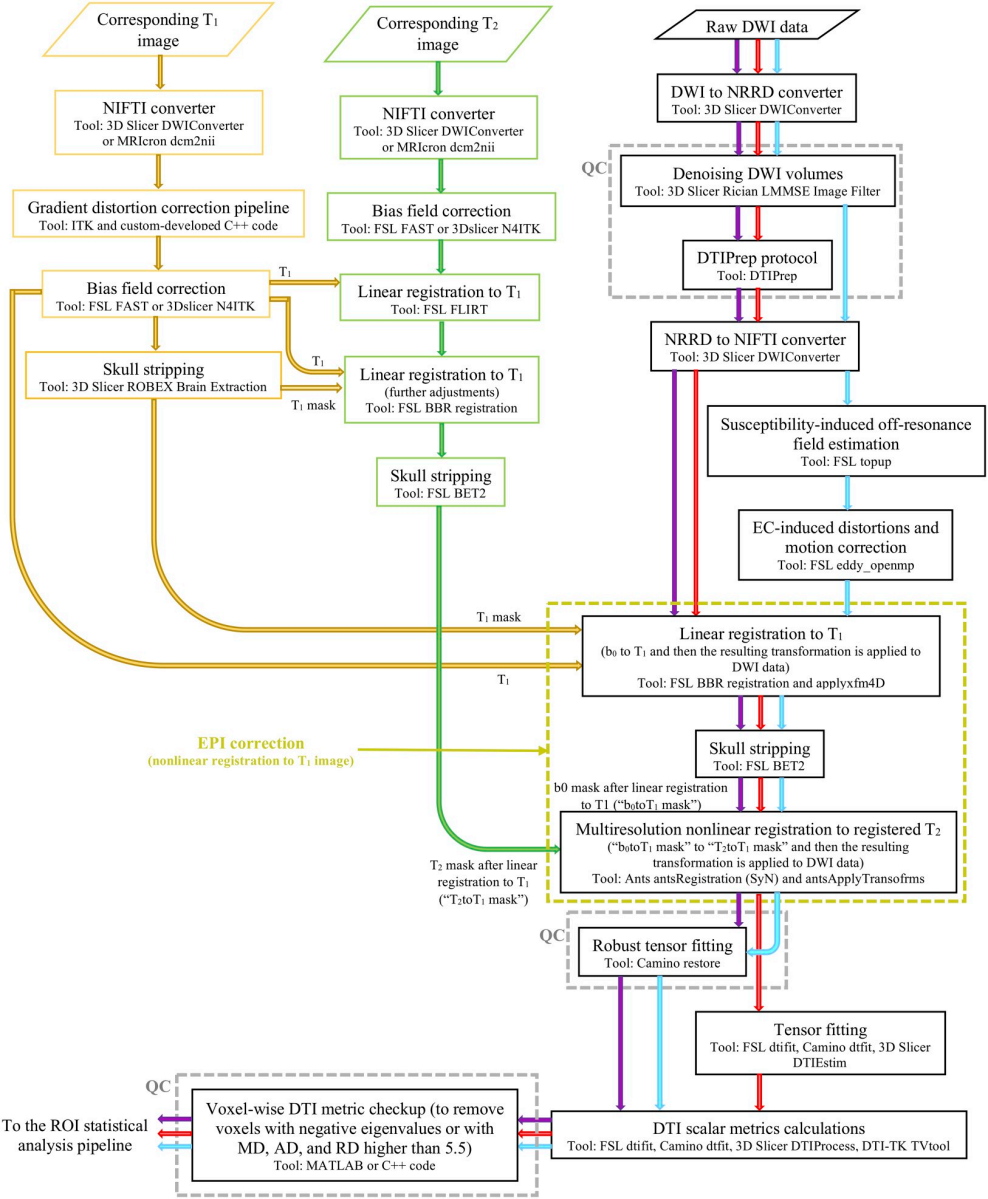
Schematic representation of the three pipelines utilized for brain DTI data processing. The T_1_- and T_2_-weighted imaging processing subpipelines are common to all DTI processing pipelines. The blue, red, and purple arrows depict the 1^st^, 2^nd^, and 3^rd^ DTI processing pipelines, respectively. T_1_ = T_1_-weighted image, T_2_ = T_2_-weighted image, b_0_ = b_0_ image.

The proposed pipelines are illustrated in Fig 1. As we used three different MR modalities, i.e. T_1_-weighted, T_2_-weighted, and diffusion images in all the proposed pipelines, we describe the subpipelines related to the processing of each MR modality as shown in Fig 1. The three proposed pipelines differ only in the DTI processing subpipelines.

#### Pipeline #1 based on robust tensor fitting

T_1_-weighted image processing subpipeline. The DTI data were registered to the native T_1_-weighted image space. This was done so that subject specific tissue segmentations could be applied to the DTI scalar maps to obtain ROI based DTI scalar metrics. Moreover, the T_1_-weighted image was used as the target of the nonlinear registration of the DWI data in the proposed DTI pipelines, to improve the susceptibility-induced EPI distortions on the diffusion data. The following image processing procedures were performed on the T1 images (Fig 1):

1. **DICOM to NIFTI conversion**. The T_1_-weighted image was converted from DICOM [49] to 3D NIFTI [50] volumes so that image processing procedures could be applied to the data. Tools such as DWI Converter in 3D Slicer software v4.8.0 [51,52] or dcm2nii in MRIcron [53] can accomplish this image transformation. In the proposed pipelines we used DWI Converter in 3D Slicer (v4.8.0).
2. **Gradient nonlinearity distortion correction**. The NIFTI T_1_-weighted image was transformed to eliminate gradient nonlinearity artifacts from the image [54] using a procedure previously described [55]. Gradient distortions alter brain geometry [54] and may reduce the accuracy of tissue segmentation. This step was performed in an attempt to reduce site-specific image distortions to improve the precision of the quantitative morphometric analyses [54].
3. **Bias field correction**. Inhomogeneity of the magnetic field within the MR scanner is inevitable due to two major factors: 1) inherent inhomogeneity of the MRI magnet, and 2) inhomogeneity of the magnetic properties of the subject [33]. This inhomogeneity leads to low spatial frequency intensity (gray value) alteration across the image known as a bias field [33]. The bias field artifacts can be eliminated from the T1-weighted image using tools such as FAST in FSL (v5.0) [33] or N4ITK in 3D Slicer (v4.8.0) [56] using random field estimation models and expectation-maximization algorithms.
4. **Skull stripping**. The brain was extracted from the skull and other tissues surrounding the brain to increase processing efficiency and improve the performance of the registration of the DWI data and T_2_-weighted images to the T_1_-weighted images. There are many different algorithms and software tools to extract the brain from the T_1_-weighted structural images. In the current study, we used the robust brain extraction tool (ROBEX) in 3D Slicer (v4.8.0) [57] for skull stripping, which provided consistent high quality brain extraction in most cases tested. It is noteworthy that the performance and quality of the image extraction tools depend on the quality of the MR images.

T_2_-weighted image processing subpipeline. The T_2_-weighted MR images were utilized in all the proposed DTI processing pipelines to improve the quality of the nonlinear registration of the DWI data (mainly the b = 0 diffusion image) to the T_1_-weighted structural image. To the best of our knowledge, the nonlinear registration of DWI data to the T_1_-weighted structural image is challenging using common registration schemes [58,59]. This stems from the fact that both the T_1_-weighted images and the b = 0 diffusion images have different tissue intensity profiles for WM, GM, and CSF, which hinders the functionality of current similarity metrics (such as mutual information) [58–60]. As the T_2_-weighted images have similar signal intensity characteristics to those found in the b = 0 image, the similarity metrics typically used in registration algorithms can more precisely assess the resemblance between these images (i.e. b = 0 and T_2_-weighted). Therefore, to improve the quality of this nonlinear registration, the b = 0 image was registered to a high quality non-EPI T_2_-weighted image as an intermediate stage for nonlinear registration of the b = 0 image to the T_1_-weighted structural image. To facilitate the nonlinear registration of the diffusion data to the T_1_-weighted image, both the b = 0 (diffusion data) and T_2_-weighted images were first linearly registered to the native T_1_-weighted structural image and then a nonlinear registration was performed between the T_2_-weighted and b = 0 images after both were linearly registered to the corresponding native T_1_-weighted image.

The T_2_-weighted image processing subpipeline was similar to the T_1_-weighted image processing subpipeline (Fig 1) except for two major differences: 1) gradient distortion correction was not applied to the T_2_-weighted image and 2) the T_2_-weighed image processing subpipeline incorporated a linear registration of the T_2_-weighted image to the T_1_-weighted structural image as explained above. To increase the accuracy of the linear registration of the T_2_-weighted image to the T_1_-weighted image, two major steps were considered in the T_2_-weighted image processing subpipeline. First, the T_2_-weighted image was linealy registered to the T_1_-weighted image using the well known linear registration tool FSL FLIRT [61,62]. Then, alignment was improved using a boundary-based registration (BBR) tool avaialble in FSL that was applied to the output of the first registration [63].

Diffusion weighted imaging processing subpipeline. The diffusion weighted imaging processing subpipeline involved a number of image processing procedures (Fig 1):

1. **DICOM to Nearly Raw Raster Data (NRRD) conversion**. We used the DWIConverter module in 3D Slicer (v4.8.0) to transform the DICOM data from the scanner to a 3D NRRD volume rather than NIFTI format. This step was performed because the Rician linear minimum mean square error (LMMSE) Image Filter Module in 3D Slicer used in the next stage of the subpipeline requires NRRD input images.
2. **Denoising DWI volumes**. DWI typically suffers from low SNR and is thus highly prone to noise artefacts [64–66]. DTI is known to be affected by Rician noise [64–66], which imposes a positive bias on the calculated voxelwise diffusion tensors [64]. Consequently, it is vital to reduce the level of Rician noise in the diffusion data. The level of the denoising must not oversmooth the data, which decreases image resolution and may reduce the ability to differentiate diverse types of cerebral tissues/lesions [67]. Therefore, we moderately denoised the DWI data using the Rician LMMSE Image Filter Module in 3D Slicer [66], with the iteration parameter of the filter set to 1. This denoising step in the DTI processing pipeline can be regarded as a QC procedure moderating the effect of the noise on the DWI data.
3. **NRRD to NIFTI converter**. After denoising, the NRRD data was converted to NIFTI format, for compatibility with most of brain DTI processing software packages including FSL and Camino. For this image conversion, we used the DWIConverter module in 3D Slicer (v4.8.0).
4. **Estimation of the susceptibility-induced off resonance field**. Off resonance spins arising from inhomogeneities of the static magnetic field, can produce artefacts in the DWI volumes that appear as unidirectional stretching or compression [68,69]. These off resonance spins occur due to magnetic susceptibility variations throughout the brain known as susceptibility-induced off resonance fields [68,69]. A systematic approach to eliminate the susceptibility-induced off resonance field from the EPI images is to collect either a magnetic field map or a second DWI dataset with reverse phase-encode (PE) direction during each specific diffusion acquisition [70]. Neither of these two options were implemented in the ONDRI DWI data acquisition, as greater scan time is needed, which is problematic for the ONDRI cohort suffering from various neurodegenerative conditions. Consequently, to minimize the effect of the susceptibility-induced artifacts on the ONDRI DWI data, we used a nonlinear registration to the T_1_-weighted structural image based on the recommendation made in the ENIGMA DTI framework (khaki dashed rectangle in Fig 1) [9,24]. Meanwhile, b = 0 images obtained on Siemens and Philips platforms as part of the DWI dataset were acquired with reverse PE direction, which was input to the well-known FSL top-up tool to estimate the susceptibility-induced off resonance field [68]. This estimated susceptibility-induced off resonance field in the absence of the DWI data with reverse PE direction was utilized as an input argument to the FSL eddy tool, to correct susceptibility-induced artefacts across the diffusion images [69]. Additional b = 0 images with reverse PE direction were not acquired on GE scanners, and hence this step was not performed (Fig 1).
5. **EC-induced distortion and motion correction**. Eddy currents induced in the magnet bore by fast gradient-switching during the diffusion weighted echo planar imaging sequence produces a time-dependent off resonance field [69]. In addition, head motion during long diffusion scans produces shifts in head position at different diffusion weightings [69]. Both artefacts are tackled in the FSL eddy tool by registering each individual gradient image to a model-free prediction of that image [69]. To increase the computational efficiency of the eddy and motion corrections procedure, we used FSL (v5.0) eddy_openmp, which is executable on multiple CPU cores using parallelization algorithms. The FSL eddy (step 5) and topup (step 4) tools used in the 1^st^ DTI processing pipeline were adopted from FSL version 5.0.9. In that version correcting slice-to-volume movement and correcting susceptibility-by-movement interactions with eddy were not available and hence were not used. These options may be used to apply appropriate eddy/motion corrections in case of DTI data with severe motion artifacts.
6. **EPI distortion correction**. As indicated previously neither a magnetic field map nor DWI data with reverse PE direction were acquired. Hence, EPI distortion correction was achieved in the presented DTI processing pipeline using a nonlinear registration to the corresponding T_1_-weighted structural images [71,72]. Registration of the DWI data to the T_1_-weighted images as described in the T_2_–weighted images processing subpipeline section was performed using a two-stage registration scheme. First, the DWI b = 0 image was linearly registered to the T_1_-weighted structural image using the FSL (v5.0) BBR tool. Then, the SyN registration algorithm implemented in the ANTs software [73,74] was utilized for nonlinear registration of the b = 0 image to the T_2_-weighted image, which was itself already transferred to the native T_1_-weighted image space using a linear registration. Prior to the SyN nonlinear registration, the skull was stripped in the DWI data to improve the quality and computational efficiency of the nonlinear registration. Skull stripping was performed in the diffusion processing subpipeline using FSL BET2 [75].
7. **Robust tensor fitting**. In this step, an automatic QC procedure was performed on the DWI data. For this purpose, we used the RESTORE algorithm in the Camino software, which detects local voxelwise outliers in the DTI data and excludes them from the final tensor estimation procedure using iteratively reweighted least-square regression [27].
8. **Calculate DTI scalar metrics**. In this step, DTI scalar metrics including FA, mean diffusivity (MD), axial diffusivity (AD), and radial diffusivity (RD) maps were calculated throughout the brain. Diverse tools such as Camino dtfit [76,77], FSL (v5.0) dtifit [78,79], and DTI-TK TVtool [80] can be used to estimate voxelwise DTI metrics. In the 2^nd^ pipeline, we used FSL (v5.0) dtifit while in the 1^st^ and 3^rd^ pipelines the DTI-TK TVtool was utilized.
9. **Voxelwise QC procedure**. At this final step the diffusion tensor in all the voxels throughout the brain was checked to obtain more reliable DTI metrics. To this end, the voxels whose diffusion tensors had at least one negative eigenvalue or whose AD, MD, or RD were higher than 5.5 were excluded from the ROI analysis [1].

#### Pipeline #2 based on the DTIPrep protocol

This pipeline is similar to Pipeline #1 except the QC procedure performed in step 7 of the DTI processing pipeline described above was removed and replaced by the DTIPrep QC protocol (v1.2.8) [13,26] after denoising (Fig 1).

**DTIPrep QC protocol**. This automatic QC protocol utilizes global rejection of the diffusion volumes affected by various DWI artefacts such as inter-slice brightness artefacts, venetian blind artefacts, eddy current distortions, and motion artefacts, beyond the threshold limits defined by the DTIPrep protocol [13]. During the DTIPrep QC procedure, all gradient volumes were linearly registered to the b = 0 image in the DWI data [13]. Moreover, in the 2^nd^ pipeline, the topup, EC, and motion correction blocks considered in pipeline #1 (i.e. steps 4 and 5) were also skipped as they were implemented inside the DTIPrep automatic QC software package [13].

#### Pipeline #3 combining the DTIPrep protocol with robust tensor estimation

In the 3^rd^ pipeline, both QC pipelines were applied (Fig 1) to detect outliers and remove them from brain DWI data.

### Evaluation of the processing error of the pipelines

Each DTI processing pipeline introduces some error during the processing of the MR images. To evaluate this error all of the proposed pipelines were utilized to process the simulated ground truth DWI data. Note that the EPI distortion correction was not included in the processing of the ground truth DWI data as the datasets did not include any EPI-related artifacts. Moreover applying the denoising Rician LMMSE filter was also omitted because the dataset did not include any noise and applying this additional filter would have introduced unnecessary artifacts.

To evaluate the processing error in each pipeline, the difference of the mean value of each DTI metric, i.e. FA, MD, AD, and RD from the ground truth values were examined in both GM and WM regions. This difference was then normalized to the corresponding ground truth mean DTI metric value to calculate the percentage error associated with each DTI metric. To compare pipeline performance, the percentage errors associated with FA and MD calculations in eight GM and WM regions including hippocampus, thalamus, temporal lobe-GM, temporal lobe-WM, parietal lobe-GM, parietal lobe-WM, frontal lobe-GM, and frontal lobe-WM were examined. For a more precise voxelwise comparison, the FA and MD of each voxel in the WM region of the brain in the subject that was used to generate the ground-truth DTI data was examined. Voxels were excluded with very small FA or MD values (FA or MD<1e-5) that were unrealistic and likely due to inaccuracies associated with the WM region mask. FA and MD values produced by the three DTI processing pipelines were compared using ANOVA. The distribution of the FA and MD values as well as the ground-truth data were also examined. The Pearson correlation coefficients between the voxelwise FA values and MD values were also calculated between each pipeline.

### Pipeline Evaluation based on the simulated artifactual DWI data

All of the proposed pipelines were also applied to four imperfect datasets: LM-20, LM-40, SM-20, and SM-40 [28,32]. Note that the EPI distortion correction was not applied because these datasets do not include EPI-related artifacts [28]. Similar to the previous section, the percentage error of the mean value of the DTI metrics from the ground truth value was measured in both GM and WM regions. To compare pipelines performance, the percentage errors associated with FA and MD calculations in eight GM and WM regions were examined.

### Evaluation of the pipelines based on in vivo ONDRI DWI data

All the proposed pipelines along with an additional DTI processing pipeline that included no quality control steps, eddy current/motion correction, or artifact/noise removal but did apply linear and non-linear registration to the T1 space (pipeline-no QC), were applied to the 20 DWI datasets from the ONDRI VCI cohort to examine pipeline performance. FA and MD measurements were compared in NAWM using ANOVA followed by post hoc tests to identify differences between the pipelines.

### Pipeline performance following denoising

To reduce Rician noise in a DWI dataset the Gradient Anisotropic Diffusion (GAD) filter [81] that is implemented in 3D Slicer was applied. This filter was tested on simulated the LM-20 dataset, which had the lowest quality among the tested datasets considering both the magnitude of motion artifacts and level of thermal noise. Parameters were chosen for the GAD filter (Conductance = 1, number of iterations = 5) to avoid blurring the edges of the image [82]. The percentage error of the DTI metrics were compared for all pipelines in WM regions between the LMMSE denoising filter (default filter, Fig 1) and the GAD denoising filter.

## Results

### Image processing

#### Ground truth dataset

**Fig 2:**
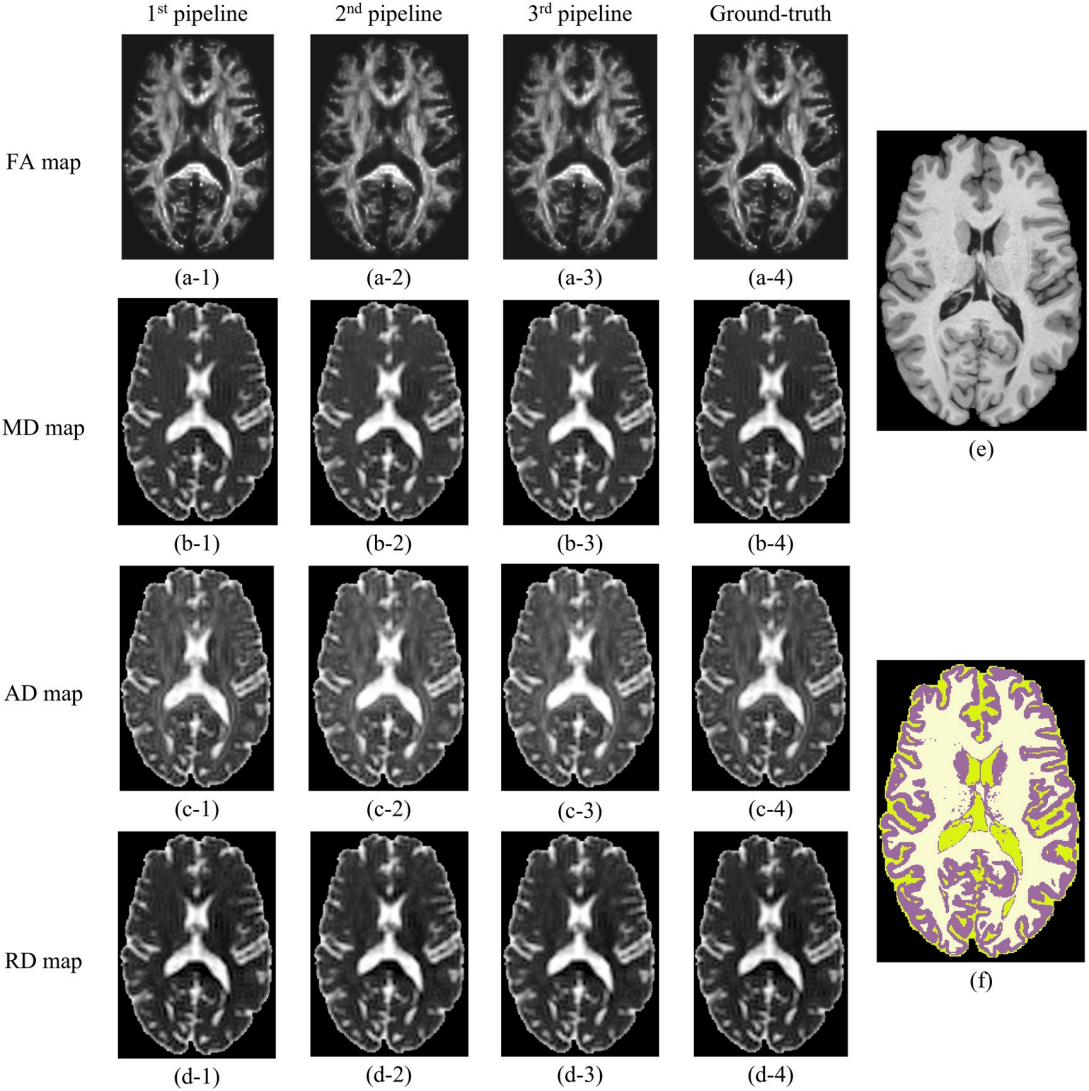
(A) FA, (B) MD, (C) AD, and (D) RD maps produced from the raw ground truth DWI data by the three DTI processing pipelines and directly from the ground truth data. First, second, and third columns correspond to the 1^st^, 2^nd^, and 3^rd^ DTI processing pipeline, respectively. The fourth column corresponds to the maps obtained directly from the ground truth data. (E) The corresponding T_1_-weighted structural image and (F) full-brain segmentation of cerebral tissues (WM, GM, and CSF) are also provided. The images were not interpolated.

All three pipelines successfully produced parametric maps of FA, MD, AD, and RD after processing the raw data without noise and artefacts that was comparable to the ground truth DTI metric maps obtained directly from ground truth data through tensor fitting without any further processing (Fig 2). The average run time for Pipeline #1 and Pipeline #3 was ~360 minutes, while Pipeline #2 took ~120 minutes to complete. Pipelines #1 and #3 likely required more time to process the ground truth data because the RESTORE algorithm consumed time (more iterations) for iterative reweighting to fulfill the goodness of fit criterion needed for successful robust tensor fitting.

#### Simulated data containing artefacts

**Fig 3:**
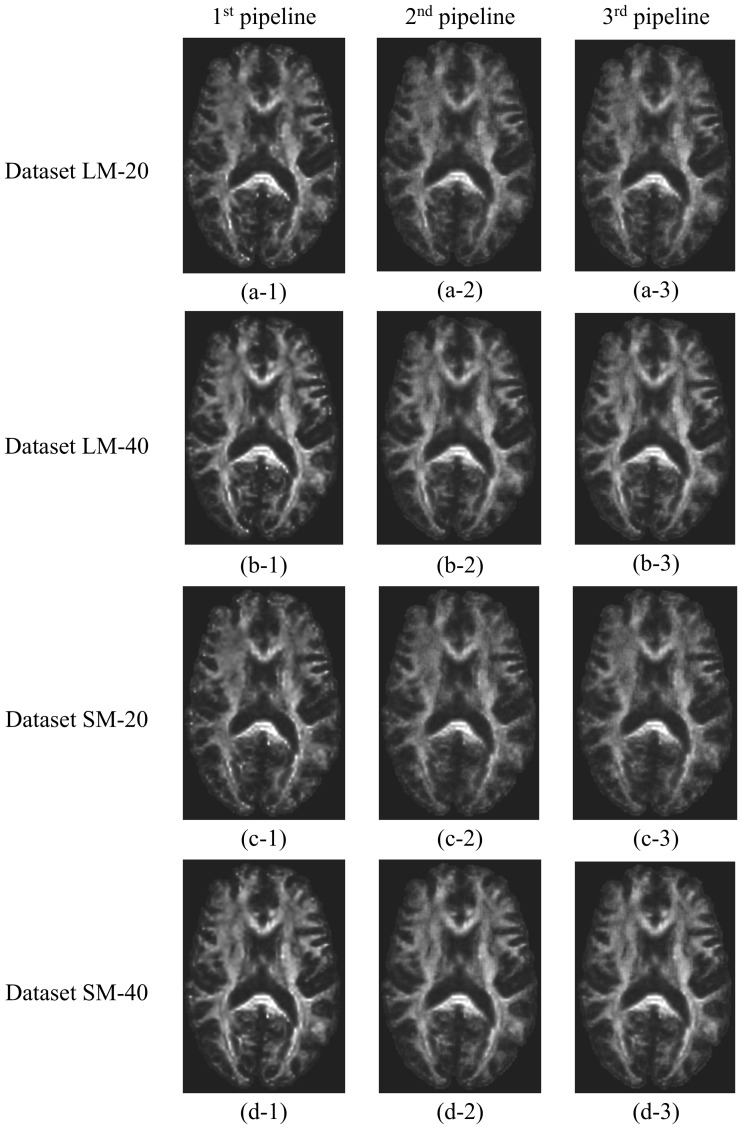
FA maps produced by the three proposed DTI processing pipelines from the DWI datasets containing artefacts. First, second, and third columns correspond to the 1^st^, 2^nd^, and 3^rd^ DTI processing pipeline, respectively. First, second, third, and forth rows correspond to datasets LM-20, LM-40, SM-20, and SM-40, respectively. The corresponding T_1_-weighted structural image and full-brain segmentation of cerebral tissues are provided in panels e and f of Fig 2, respectively. The images were not interpolated.

**Fig 4:**
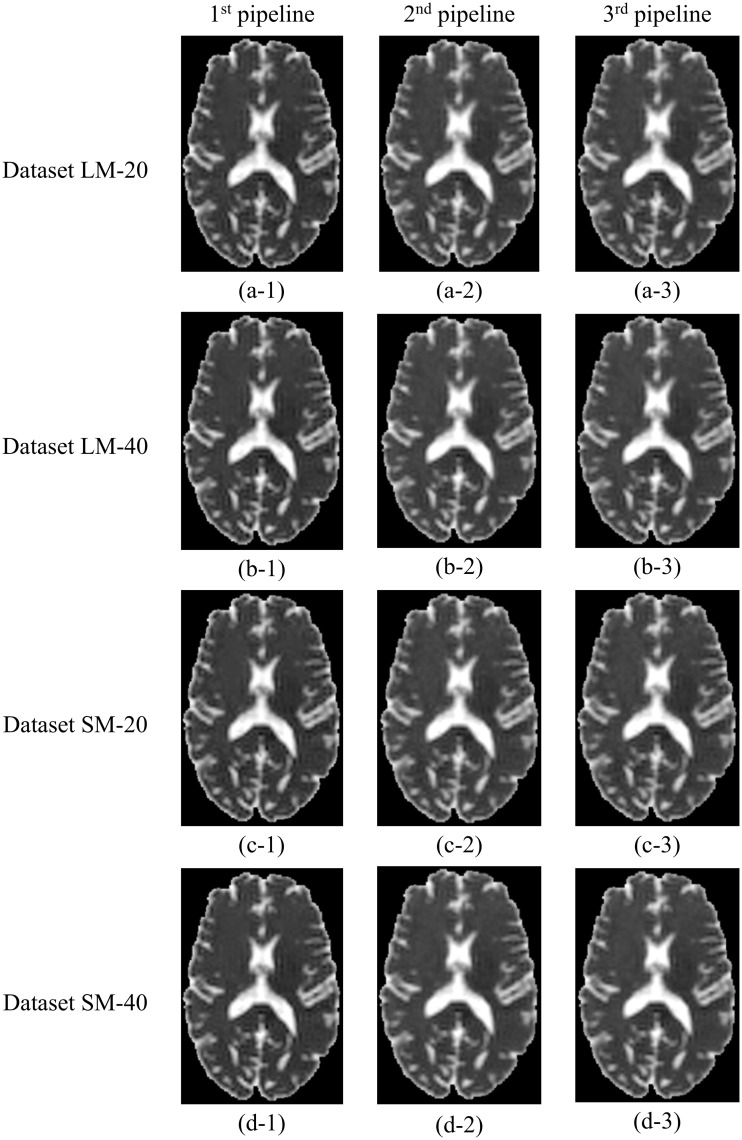
MD maps produced by the three proposed DTI processing pipelines from the DWI datasets containing artefacts. First, second, and third columns correspond to the 1^st^, 2^nd^, and 3^rd^ DTI processing pipeline, respectively. First, second, third, and forth rows correspond to datasets LM-20, LM-40, SM-20, and SM-40, respectively. The corresponding T_1_-weighted structural image and full-brain segmentation of cerebral tissues are provided in panels e and f of Fig 2, respectively. The images were not interpolated.

All three pipelines successfully produced parametric maps of FA, MD, AD, and RD after processing the simulated DWI datasets containing artefacts: LM-20, LM-40, SM-20, and SM-40. The FA and MD maps corresponding to these datasets are shown in Figs 3 and 4, respectively. These maps are qualitatively similar to the ground truth FA (Figs 2a–4) and MD (Figs 2b–4) maps, showing similar patterns of contrast. The average run time per dataset was ~120 minutes for all pipelines.

### Processing error of the proposed DTI pipelines

**Fig 5:**
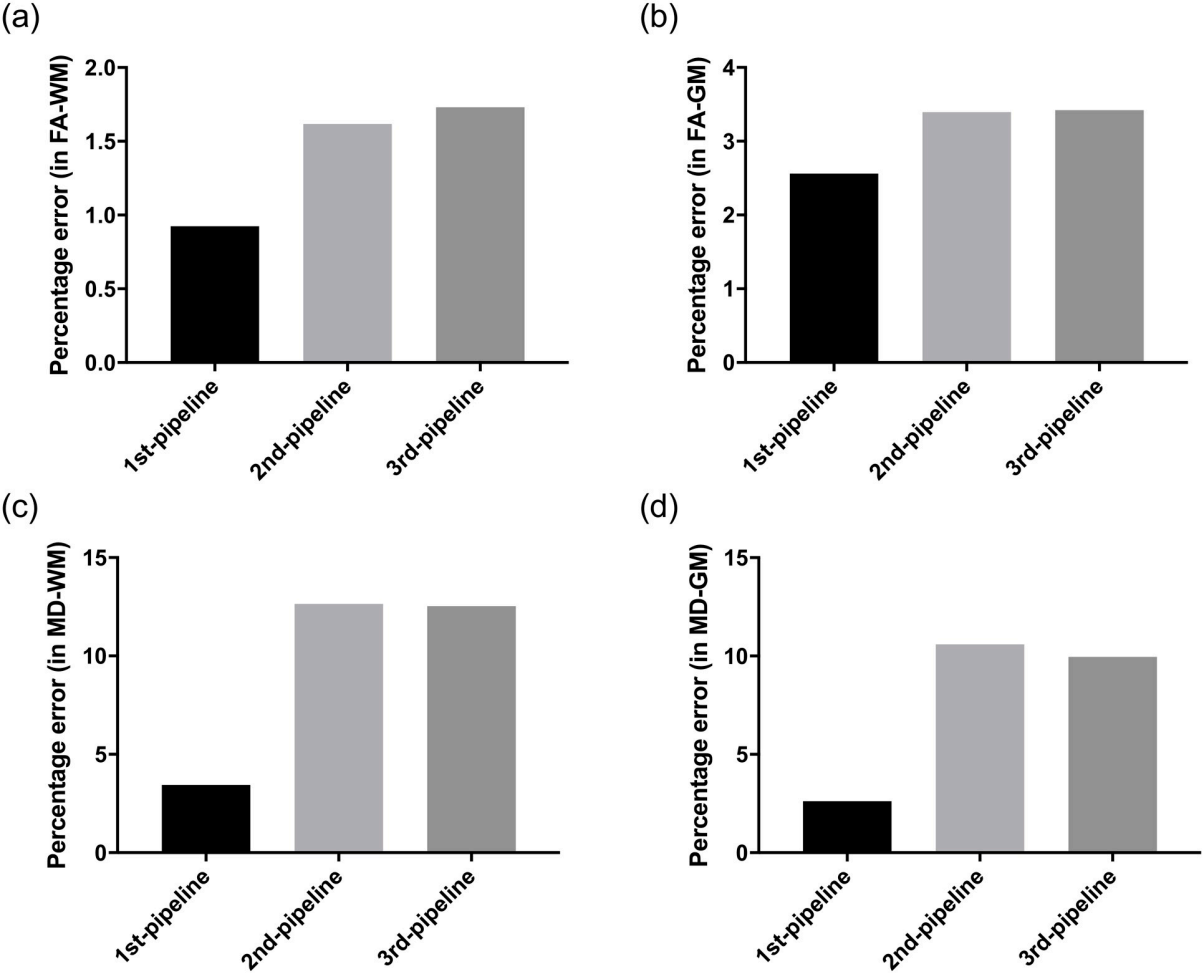
Processing error associated with the pipelines for FA calculation in (A) WM and in (B) GM, and MD calculation in (C) WM and in (D) GM.

The differences in the measured FA and MD values associated with each pipeline when processing the DWI data without noise are provided in Fig 5. Considering FA and MD in both WM and GM regions, Pipeline #1 produced the most accurate FA and MD values.

**Fig 6:**
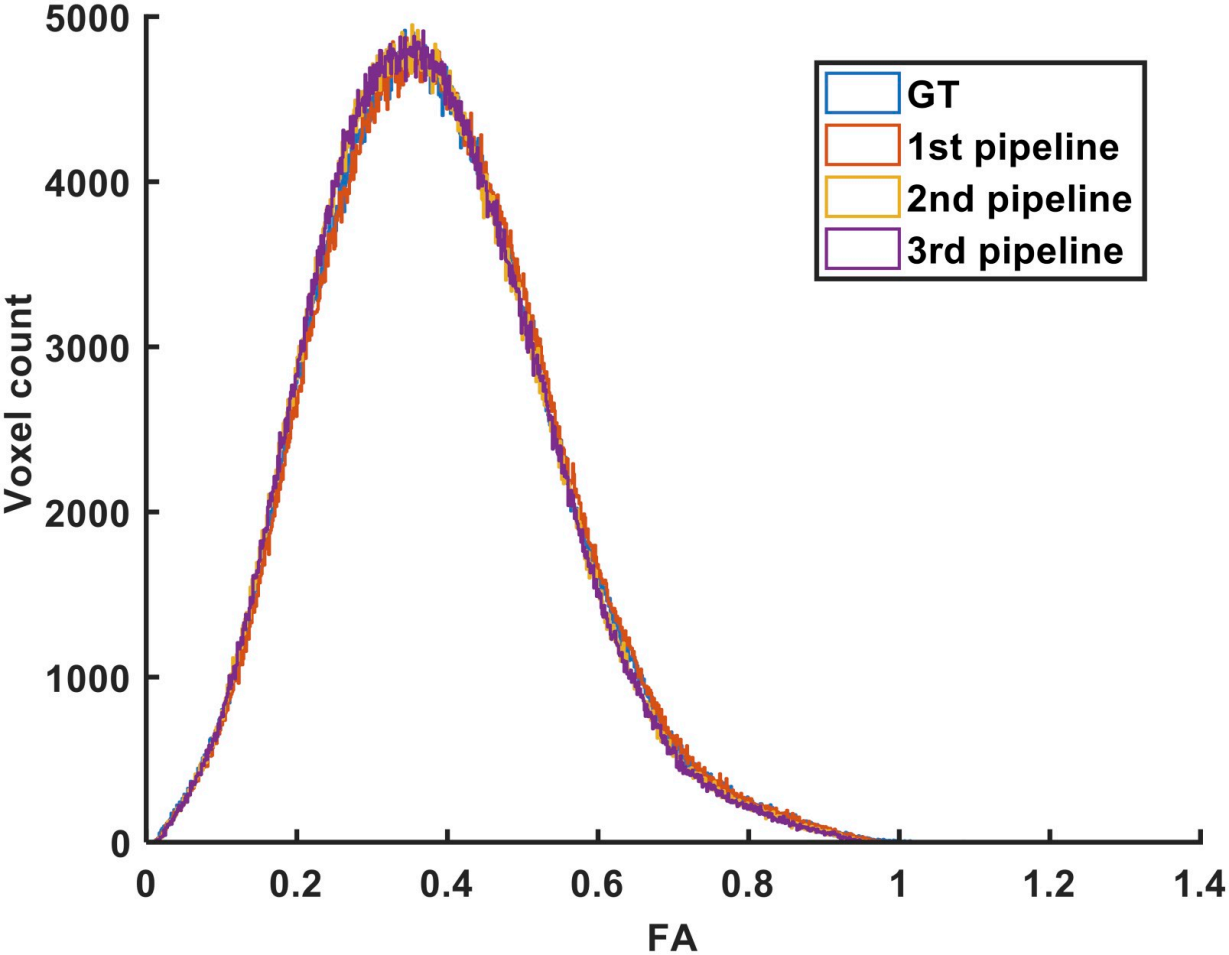
Distribution of FA values in the WM region generated by the three pipelines after processing the ground-truth data.

Histograms describing the voxelwise distribution of FA values in the WM region obtained using the three pipelines and the ground-truth data are provided in Fig 6. Despite a statistically significant difference between the pipelines (ANOVA, p<0.05) little difference is observed in the distribution of FA values.

The Pearson correlation coefficient between the FA values in each voxel obtained with the 1^st^ and 2^nd^ pipelines, 1^st^ and 3^rd^ pipelines, and 2^nd^ and 3^rd^ pipelines were 0.939, 0.940, and 0.998, respectively. The scatter plots along with the corresponding least squares regression lines are provided in S1 to S3 Figs as supporting information.

**Fig 7:**
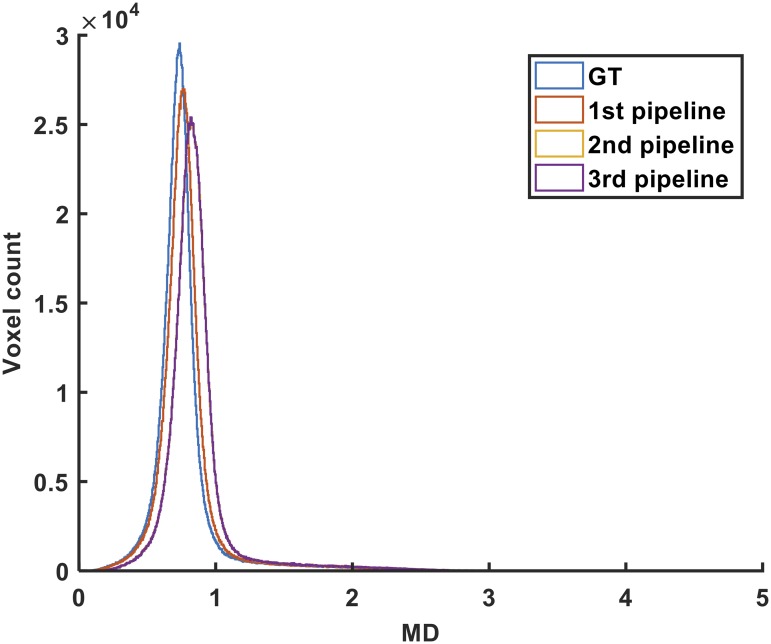
Distribution of the MD values in the WM region generated by the three pipelines after processing of the ground-truth data.

Histograms describing the voxelwise distribution of the MD values in the WM region obtained using the three pipelines and the ground-truth data are provided in Fig 7. There was also a significant difference between the MD values obtained by the three pipelines (ANOVA, p<0.05).

The Pearson correlation coefficient between the MD values in each voxel obtained with the 1^st^ and 2^nd^ pipelines, 1^st^ and 3^rd^ pipelines, and 2^nd^ and 3^rd^ pipelines were 0.978, 0.979, and 0.999, respectively. The scatter plots along with the corresponding least squares regression lines related to these correlation analyses are also provided in S4 to S6 Figs as supporting information.

### Pipeline performance when processing DWI data containing artefacts

**Fig 8:**
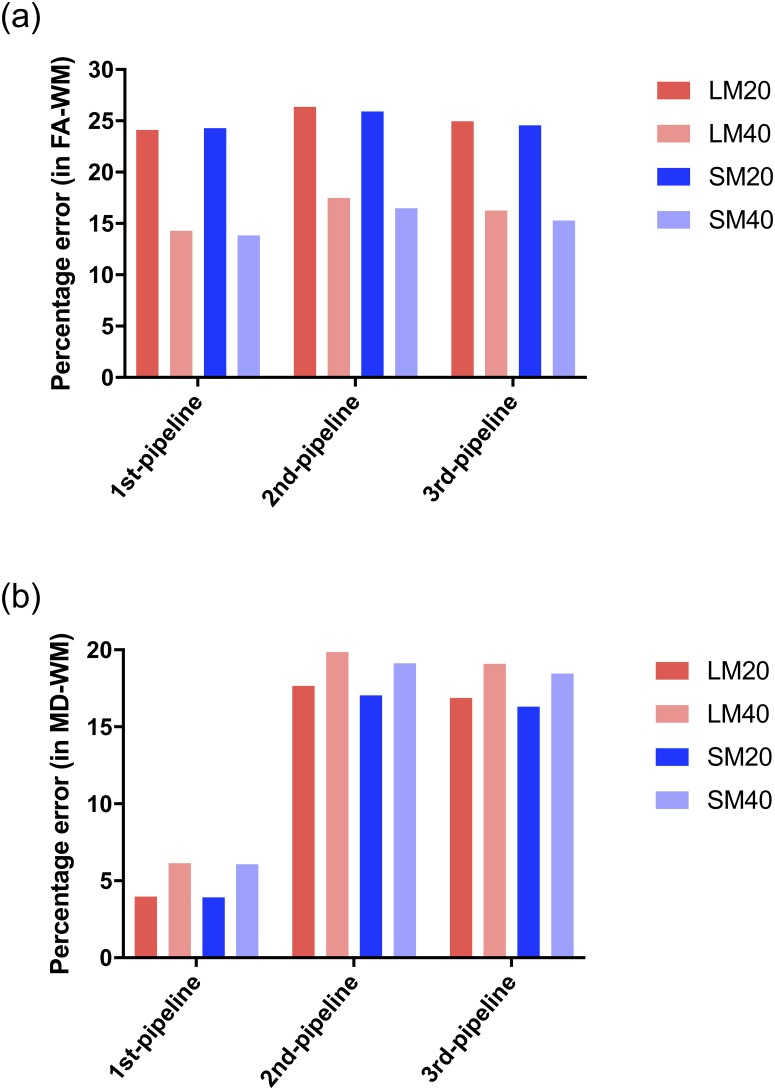
Percentage error associated with each pipeline when processing data containing artefacts. (A) shows FA in WM, (B) shows MD in WM region.

The differences in the measured FA and MD values in WM associated with each pipeline when processing the datasets containing artefacts is provided in Fig 8. In the case of FA, (Fig 8a), the percentage error did not differ significantly among the pipelines. In the case of MD, (Fig 8b), the percentage error associated with Pipeline #1 was lower than the other two pipelines.

### Performance of the pipelines in processing in vivo ONDRI DWI data

**Fig 9:**
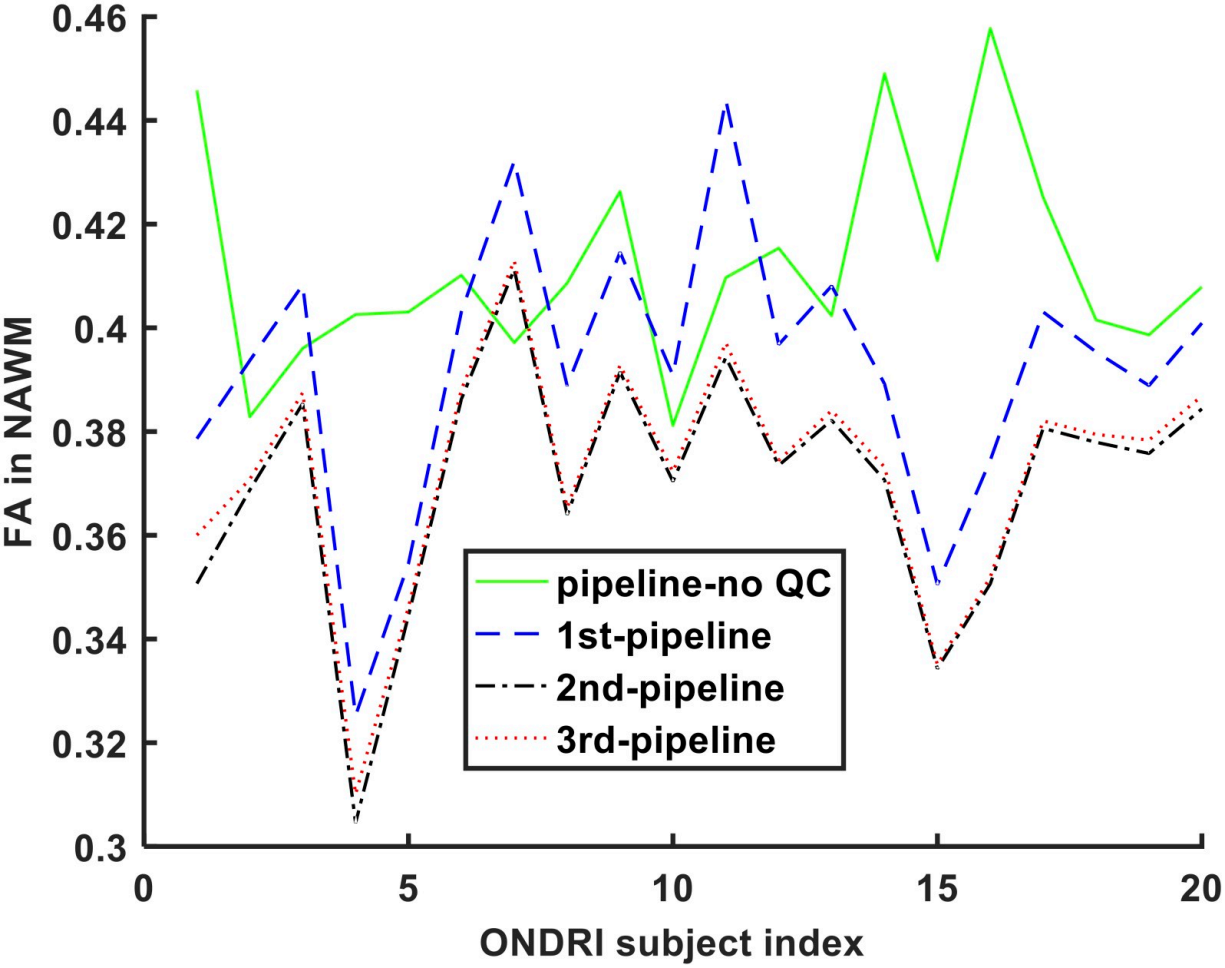
FA measurements in NAWM from 20 ONDRI VCI subjects.

There was a significant difference in FA values measured by the pipelines in NAWM across the 20 ONDRI VCI subjects (p<0.01). Multiple comparison post hoc tests also showed that all pipelines differed significantly from each other (p<0.05) except pipeline-no QC and the 1^st^ pipeline (p = 0.051), the 1^st^ and 3^rd^ pipelines (p = 0.051), and the 2^nd^ and 3^rd^ pipelines (p = 0.989). FA measurement variations in NAWM is also depicted in Fig 9.

**Fig 10:**
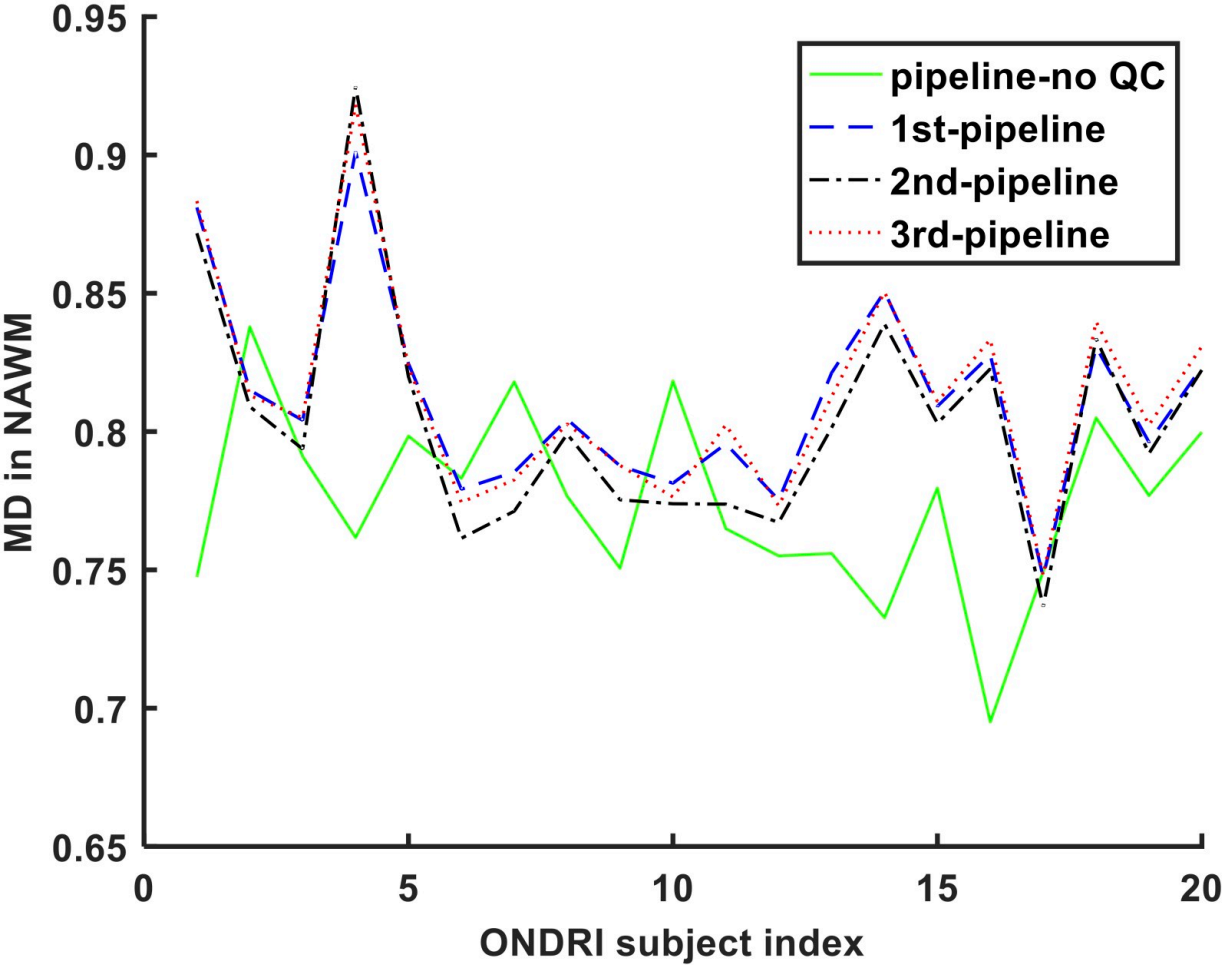
MD measurements in NAWM from 20 ONDRI VCI subjects.

There was a significant difference in MD values measured by the pipelines in NAWM across the 20 ONDRI VCI subjects (p<0.01). Multiple comparison post hoc tests also showed that all pipelines did not differ significantly from each other (p>0.05), except the pipeline with no QC and the 1^st^ pipeline (p<0.05) and the pipeline with no QC and the 3^rd^ pipeline (p<0.05). It should be noted that the three proposed pipelines with diverse QC procedures (DTI-Prep and RESTORE) did not differ significantly with regard to MD measurement performance. MD measurements in NAWM made by all four pipelines are shown in Fig 10.

### Performance of the pipelines with an alternative denoising filter

**Fig 11:**
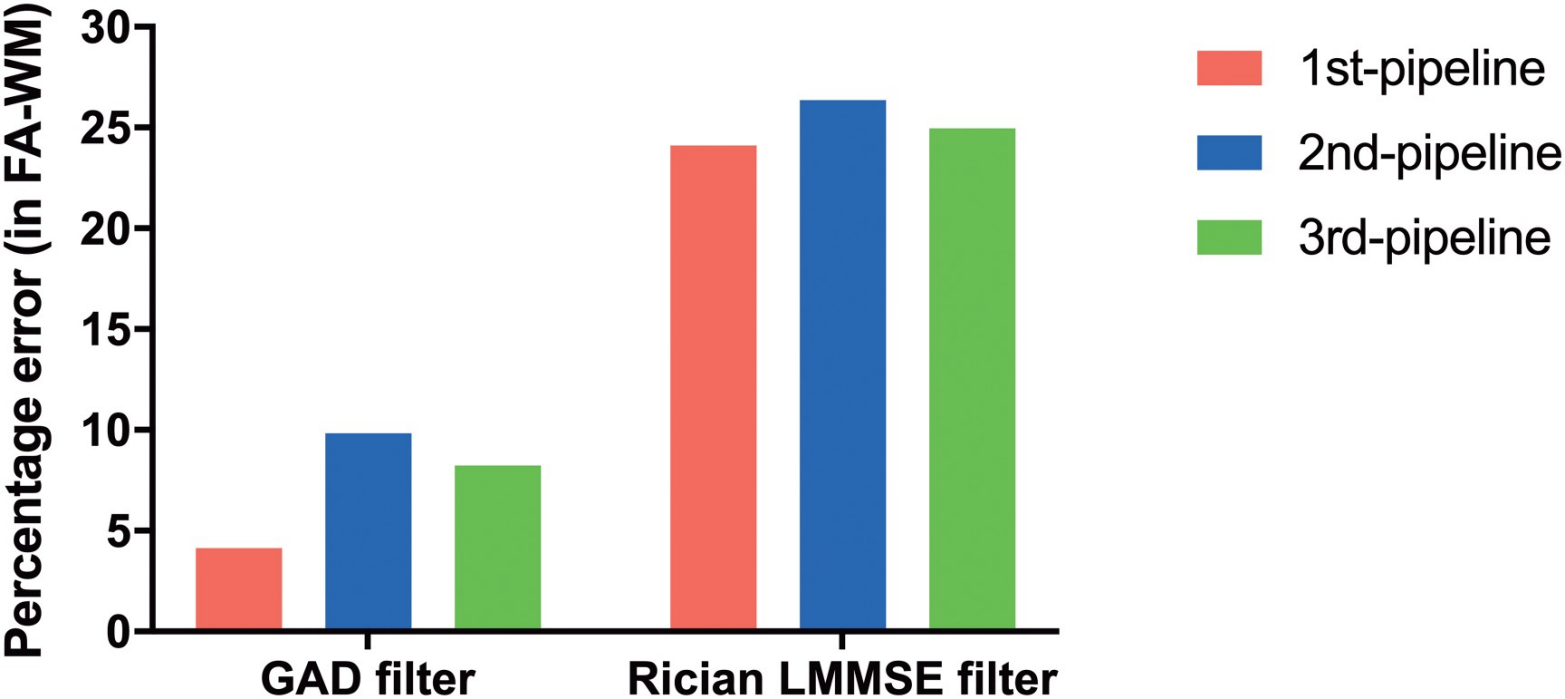
Percentage error in calculation of FA in WM for the dataset LM-20 when using the gradient anisotropic diffusion (GAD) filter and Rician linear minimum mean square error (LMMSE) filter. All pipelines produced lower percentage error with the GAD filter.

**Fig 12:**
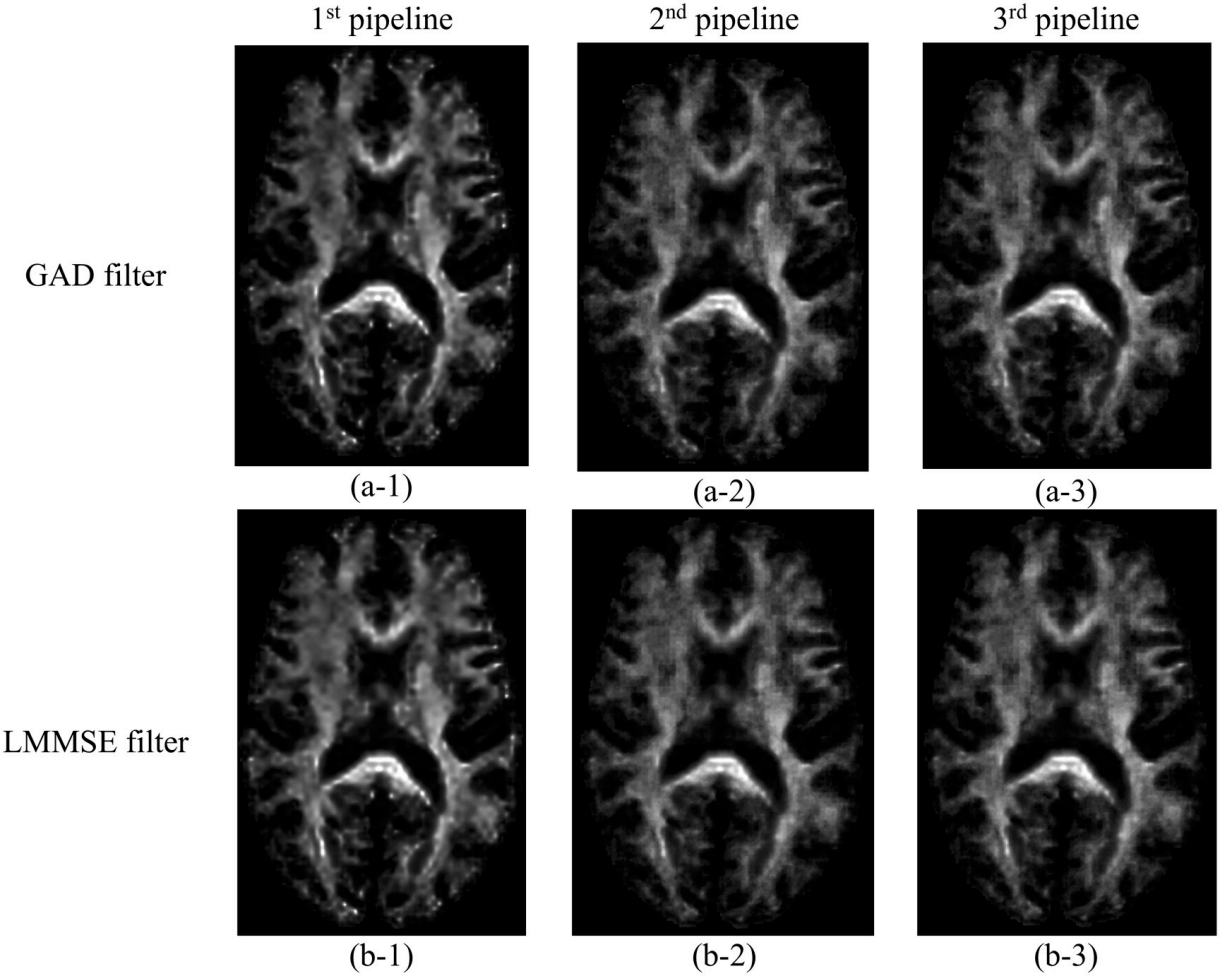
FA maps for the dataset LM-20 obtained by different pipelines when using the gradient anisotropic diffusion (GAD) filter (first row) and Rician linear minimum mean square error (LMMSE) filter (second row). First, second, and third columns correspond to the 1^st^, 2^nd^, and 3^rd^ DTI processing pipeline, respectively. The images were not interpolated.

The percentage error of the proposed pipelines for calculation of FA in WM when using the GAD filter in comparison to the LMMSE filter is depicted in Fig 11. For the purpose of qualitative comparisons the FA maps generated by the pipelines when using these two different filters were also presented in Fig 12. The use of the GAD filter increased the accuracy associated with all pipelines.

## Discussion

In this study, three different automated pipelines are presented for the analysis of the multisite ONDRI brain DTI data, each complying with the ENIGMA framework. The pipelines were designed by uniquely combining multiple image processing software tools to robustly and efficiently measure DTI metrics in the brain. In the proposed pipelines, three different QC procedures were utilized including robust tensor fitting (1^st^ pipeline), the DTIPrep protocol (2^nd^ pipeline), and the combination of both methods (3^rd^ pipeline), to determine which produced optimal estimation of DTI metrics. It is noteworthy that based on the sequential application of these two QC procedures (DTIPrep and RESTORE) in the 3^rd^ pipeline we expect that the overlap of the outliers detected by these algorithms would be minimal since these two algorithms affect the diffusion data on two different levels: the gradient volume level (in DTIPrep), and the voxel level (in RESTORE). These pipelines were applied to simulated DTI data to measure the processing error of the pipelines, to simulated DTI data containing artefacts to validate and compare the performance of the pipelines, and to 20 in vivo datasets from people with vascular cognitive impairment, to examine performance of the pipelines in a multi-site study.

All the pipelines successfully produced maps of the DTI metrics (FA, MD, AD, and RD maps) from the simulated ground-truth data and the simulated data containing noise and artifacts, the latter were qualitatively similar to the ground truth maps. The 1^st^ pipeline produced the most accurate DTI metric values when compared to the ground truth, particularly for MD. Histograms showing the voxel wise distribution of FA and MD in NAWM obtained using the proposed pipelines (Figs 6 and 7) confirm that the performance of the three pipelines were similar for FA. Greater differences were observed between the 1^st^ pipeline and the two other pipelines for MD.

The proposed pipelines also showed performance differences when tested in simulated datasets containing eddy current induced artifact, and different levels of motion artefacts and thermal noise. The performance of the pipelines was very similar for the calculation of FA in WM. However Pipeline #1 demonstrated the greatest accuracy in calculation of both FA and MD in WM (Fig 6). Both panels in Fig 8 also support the idea that the proposed pipelines successfully handle motion artifacts. In addition, noise had the expected effect on the calculation of DTI metrics. Specifically, with a higher level of noise (SNR = 20) there was a greater percentage error in DTI metrics calculations compared to lower noise levels (SNR = 40). Therefore we decided to test the effect of a denoising filter (GAD filter) in all the pipelines for the analysis of the artifactual dataset LM-20. As shown in Fig 11, all the pipelines presented considerably greater accuracy compared to the ground truth FA using the GAD filter in comparison with the Rician LMMSE filter used by default in the pipelines. This confirms that all the pipelines have the capacity to handle different levels of noise provided that an appropriate denoising filter is applied to the raw DWI data during processing.

The pipelines containing QC procedures were also used to analyze DWI datasets from 20 ONDRI VCI subjects and produced different results compared to the pipeline with no QC. The average FA value in the NAWM region measured in the current study was 0.39 ± 0.03. This value is in agreement with previously published measurements of average FA in WM regions and specific WM tracts in VCI subjects [83–85]. Specifically, the average FA value in the WM of VCI subjects was previously reported as 0.41 ± 0.026 [83]. All three pipelines performed similarly with regard to MD measurements while only the 2^nd^ and 3^rd^ pipelines performed similarly with respect to FA measurements. The 3^rd^ pipeline was the most comprehensive as it included a combination of two different QC procedures and produced the highest precision (i.e., lower STD) for FA calculation in NAWM (Fig 9). High measurement precision is an important feature for the analysis of human cohort data where the intention is to detect pathological microstructural alterations among different neurodegenerative conditions towards more effective diagnosis and treatment of these diseases.

### Study limitations

In the current study, we registered the DTI data to the high resolution structural image before tensor fitting, which is consistent with the well-known ENIGMA protocol [23,24,86]. The primary reason was that reverse phase encode direction DTI data was not available as part of the ONDRI DTI data acquisition, and hence registering to the native T1 space provided a major benefit to diminish EPI-related artefacts (distortions). The pipelines in the current manuscript were developed for the calculation of scalar DTI metrics. These metrics are expected to be minimally affected by issues arising from signal misorientation, which are more relevant for tractography applications. The current study utilized the BBR approach to directly register DTI to the native T1-weighted image. An alternative approach is to first register the DTI data to the corresponding T2-weighted image using the BBR method followed by a linear registration to the T1-weighted image as previously described [87]. However, this additional linear registration would further interpolate the DTI data, which may not be preferred but could be considered as an alternative approach if the direct registration to the native T1 space was unsatisfactory.

## Conclusions

Three different automated DTI processing pipelines, complying with the ENIGMA framework were fully implemented and then evaluated using simulated ground truth and artifact containing DWI datasets as well as multisite DTI data from ONDRI. These unique pipelines were created by optimizing and connecting components from seven different image processing software packages. The major difference between the proposed pipelines was in the approach to quality control. All the pipelines produced similar FA measurements while the 1^st^ pipeline incorporating the RESTORE algorithm produced more accurate MD measurements. The performance of the 2nd and 3rd pipelines was very similar for both FA and MD measurements. Based on the performance of the pipelines in the analysis of the DTI data from the ONDRI database and the observed DTI metric measurement precision, we conclude that the pipeline that combined both the DTIPrep and RESTORE algorithms is more comprehensive and hence recommended for the analysis of multisite DTI data.

## Supporting information

S1 Table ONDRI Subject IDs and Scan Dates related to the vascular cognitive impairment (VCI) subjects used in this study. (DOCX)

S2 Table Percentage error in FA produced by each pipeline when processing the ground-truth data and the simulated datasets with artifacts. GT = ground-truth.
(DOCX)

S3 Table Percentage error in MD produced by each pipeline when processing the ground-truth data and the simulated datasets with artifacts. GT = ground-truth.
(DOCX)

S1 Fig Scatter plot along with least squares fitted line representing correlation between WM FA measurement in the 1^st^ and 2^nd^ pipelines when they were utilized for processing ground-truth data (β = 0.939). (DOCX)

S2 Fig Scatter plot along with least squares fitted line representing correlation between WM FA measurement in the 1^st^ and 3^rd^ pipelines when they were utilized for processing ground-truth data (β = 0.940). (DOCX)

S3 Fig Scatter plot along with least squares fitted line representing correlation between WM FA measurement in the 2^nd^ and 3^rd^ pipelines when they were utilized for processing ground-truth data (β = 0.998). (DOCX)

S4 Fig Scatter plot along with least squares fitted line representing correlation between WM MD measurement in the 1^st^ and 2^nd^ pipelines when they were utilized for processing ground-truth data (β = 0.978). (DOCX)

S5 Fig Scatter plot along with least squares fitted line representing correlation between WM MD measurement in the 1^st^ and 3^rd^ pipelines when they were utilized for processing ground-truth data (β = 0.979). (DOCX)

S6 Fig Scatter plot along with least squares fitted line representing correlation between WM MD measurement in the 2^nd^ and 3^rd^ pipelines when they were utilized for processing ground-truth data (β = 0.999). (DOCX)
