# Supplementary material for: Comparison of quality control methods for automated diffusion tensor imaging analysis pipelines
Source: PLoS One. 2019 Dec 20;14(12):e0226715. doi: 10.1371/journal.pone.0226715 (PMC6924651; doi:10.1371/journal.pone.0226715)
Supplement: S1 Table — (DOCX) [file pone.0226715.s001.docx]

| **ONDRI Subject ID** | **Scan Date** |
| --- | --- |
| OND01_HGH_5017 | 2015JUL08 |
| OND01_HGH_5027 | 2016FEB02 |
| OND01_LHS_5009 | 2014NOV28 |
| OND01_LHS_5012 | 2015MAR06 |
| OND01_LHS_5018 | 2015MAY15 |
| OND01_SBH_5004 | 2014NOV04 |
| OND01_SBH_5008 | 2015JAN23 |
| OND01_SBH_5013 | 2015FEB24 |
| OND01_SBH_5027 | 2016APR26 |
| OND01_SMH_5004 | 2015SEP09 |
| OND01_SMH_5009 | 2015DEC02 |
| OND01_SMH_5019 | 2016MAY30 |
| OND01_TBR_5006 | 2016MAY03 |
| OND01_TOH_5020 | 2014DEC04 |
| OND01_TOH_5024 | 2015MAY22 |
| OND01_TOH_5028 | 2015DEC22 |
| OND01_TWH_5003 | 2014DEC11 |
| OND01_TWH_5012 | 2015MAY14 |
| OND01_TWH_5016 | 2015JUN01 |
| OND01_TWH_5019 | 2015AUG10 |
