# Supplementary material for: Comparison of quality control methods for automated diffusion tensor imaging analysis pipelines
Source: PLoS One. 2019 Dec 20;14(12):e0226715. doi: 10.1371/journal.pone.0226715 (PMC6924651; doi:10.1371/journal.pone.0226715)
Supplement: S2 Table — GT = ground-truth. (DOCX) [file pone.0226715.s002.docx]

|  | **Dataset** | **Hippocampus** | **Thalamus** | **Temporal lobe-GM** | **Temporal lobe-WM** | **Parietal lobe-GM** | **Parietal lobe-WM** | **Frontal lobe-GM** | **Frontal lobe-WM** |
| --- | --- | --- | --- | --- | --- | --- | --- | --- | --- |
| **1^st^ pipeline** | **GT** | 0.4 | 1.3 | 1.6 | 1.4 | 1.8 | 1.8 | 1.9 | 1.7 |
|  | **LM-20** | 17.6 | 19.1 | 17 | 16.8 | 13.8 | 13 | 20.4 | 20.3 |
|  | **LM-40** | 11.8 | 12.7 | 10.1 | 10.1 | 8.7 | 8.8 | 12.5 | 12.5 |
|  | **SM-20** | 20.4 | 20.1 | 17 | 17 | 14.3 | 13.6 | 20.3 | 20.7 |
|  | **SM-40** | 10.1 | 11.2 | 10.2 | 10.7 | 8.9 | 9 | 11.9 | 11.7 |
| **2^nd^ pipeline** | **GT** | 0.9 | 0.9 | 0.9 | 1 | 1.6 | 1.8 | 0.1 | 0 |
|  | **LM-20** | 16.6 | 20.8 | 18.2 | 17.9 | 14.9 | 13.4 | 21.9 | 21.8 |
|  | **LM-40** | 10.8 | 14.8 | 11.5 | 11.5 | 9.5 | 8.7 | 14.4 | 14.5 |
|  | **SM-20** | 19.5 | 20.2 | 17.6 | 17.7 | 14.5 | 13.5 | 21 | 21.2 |
|  | **SM-40** | 11 | 12.5 | 11 | 11.3 | 9.1 | 8.5 | 12.9 | 12.4 |
| **3^rd^ pipeline** | **GT** | 0.7 | 0.8 | 0.9 | 1.1 | 1.2 | 1.5 | 0.2 | 0.1 |
|  | **LM-20** | 14.8 | 19.2 | 16.8 | 16.5 | 13.7 | 12.2 | 20.3 | 20.2 |
|  | **LM-40** | 10 | 13.3 | 10.1 | 10 | 8.4 | 7.4 | 13 | 13.1 |
|  | **SM-20** | 17.8 | 18.8 | 16.3 | 16.3 | 13.4 | 12.3 | 19.4 | 19.6 |
|  | **SM-40** | 9.8 | 11.1 | 9.7 | 10 | 8.1 | 7.4 | 11.5 | 11.1 |
