# Supplementary material for: Comparison of quality control methods for automated diffusion tensor imaging analysis pipelines
Source: PLoS One. 2019 Dec 20;14(12):e0226715. doi: 10.1371/journal.pone.0226715 (PMC6924651; doi:10.1371/journal.pone.0226715)
Supplement: S3 Table — GT = ground-truth. (DOCX) [file pone.0226715.s003.docx]

|  | **Dataset** | **Hippocampus** | **Thalamus** | **Temporal lobe-GM** | **Temporal lobe-WM** | **Parietal lobe-GM** | **Parietal lobe-WM** | **Frontal lobe-GM** | **Frontal lobe-WM** |
| --- | --- | --- | --- | --- | --- | --- | --- | --- | --- |
| **1^st^ pipeline** | **GT** | 3.8 | 3.2 | 2.7 | 2.7 | 2.3 | 2.6 | 2.7 | 2.7 |
|  | **LM-20** | 6.1 | 6 | 5.1 | 5.3 | 5.4 | 5.5 | 5.6 | 5.6 |
|  | **LM-40** | 6.9 | 6.5 | 5.9 | 6 | 6.4 | 6.3 | 5.7 | 5.5 |
|  | **SM-20** | 6.2 | 6 | 5.3 | 5.6 | 5.6 | 5.8 | 5.2 | 5.4 |
|  | **SM-40** | 6.4 | 6.2 | 5.9 | 6.2 | 6 | 6.1 | 5.6 | 5.7 |
| **2^nd^ pipeline** | **GT** | 11.2 | 9.7 | 9.5 | 9.6 | 9.2 | 9.3 | 9.3 | 9.2 |
|  | **LM-20** | 15.1 | 14.7 | 13.3 | 13.6 | 13.6 | 13.3 | 13.5 | 13.4 |
|  | **LM-40** | 16.7 | 15.4 | 14.2 | 14.5 | 14.5 | 14.3 | 14.3 | 14 |
|  | **SM-20** | 15.6 | 14 | 13.2 | 13.7 | 13.3 | 13.2 | 12.8 | 12.8 |
|  | **SM-40** | 16 | 14.6 | 14 | 14.5 | 13.8 | 14.1 | 13.5 | 13.4 |
| **3^rd^ pipeline** | **GT** | 10.6 | 9.5 | 8.8 | 9.2 | 7.1 | 8.1 | 9 | 9 |
|  | **LM-20** | 14.6 | 13.9 | 12.4 | 12.7 | 11.9 | 12.3 | 12.8 | 12.7 |
|  | **LM-40** | 15.3 | 14.6 | 13.1 | 13.5 | 12 | 12.8 | 13.6 | 13.3 |
|  | **SM-20** | 14.9 | 13.4 | 12.3 | 12.9 | 11.5 | 12.2 | 12.2 | 12.3 |
|  | **SM-40** | 15 | 14 | 13 | 13.6 | 11.7 | 12.8 | 12.9 | 12.8 |
