# Supplementary figures and images for: Comparison of quality control methods for automated diffusion tensor imaging analysis pipelines
Source: PLoS One. 2019 Dec 20;14(12):e0226715. doi: 10.1371/journal.pone.0226715 (PMC6924651; doi:10.1371/journal.pone.0226715)

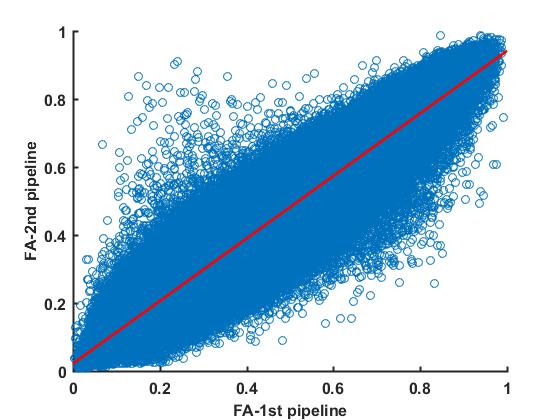

Supplement: S1 Fig — (DOCX) [file pone.0226715.s004.docx]

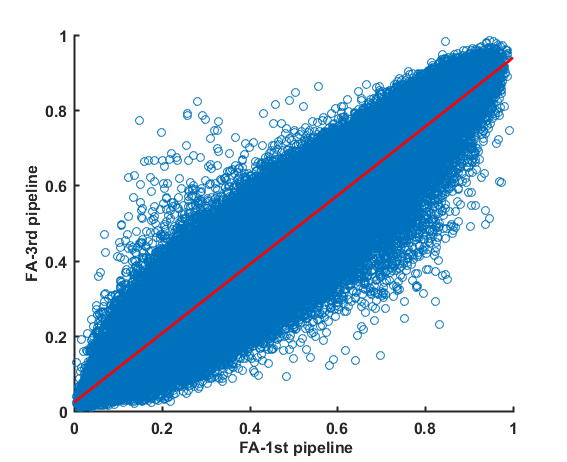

Supplement: S2 Fig — (DOCX) [file pone.0226715.s005.docx]

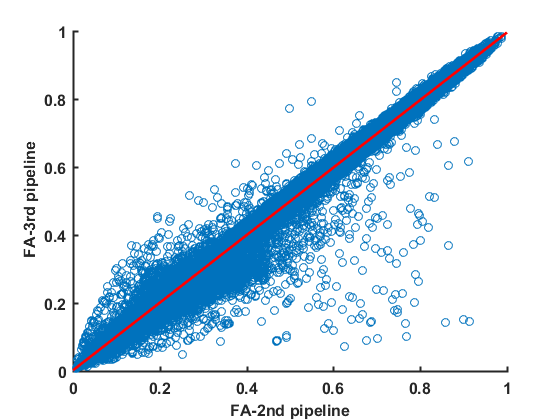

Supplement: S3 Fig — (DOCX) [file pone.0226715.s006.docx]

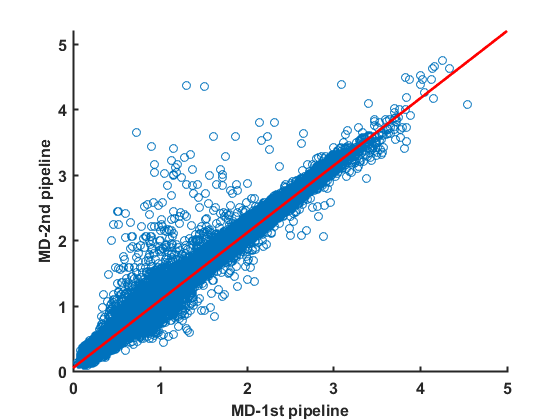

Supplement: S4 Fig — (DOCX) [file pone.0226715.s007.docx]

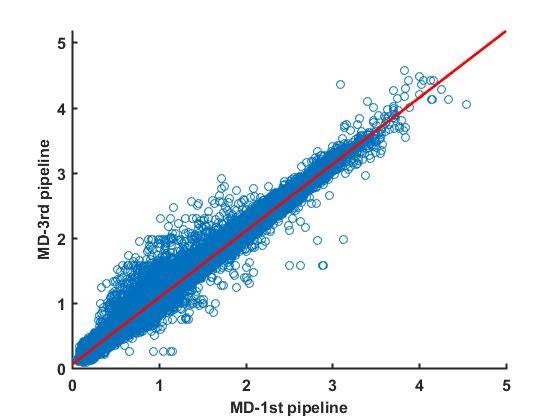

Supplement: S5 Fig — (DOCX) [file pone.0226715.s008.docx]

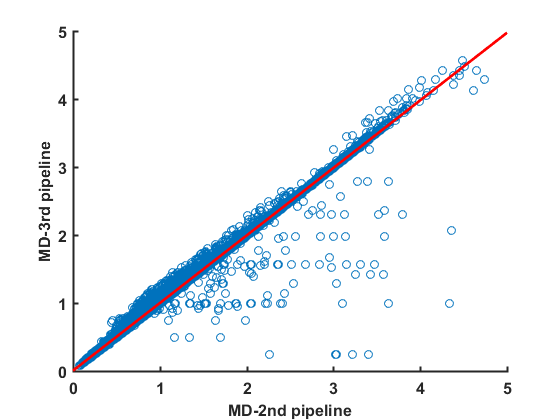

Supplement: S6 Fig — (DOCX) [file pone.0226715.s009.docx]
